# Adverse Consequences of Unmet In-Home Mobility Care Needs and Risk of Hospital Stay Among Older Adults in the United States

**DOI:** 10.1093/geroni/igaa057.142

**Published:** 2020-12-16

**Authors:** Shalini Sahoo

**Affiliations:** University of Maryland, Baltimore, Elkridge, Maryland, United States

## Abstract

Mobility limitations are the most prevalent late life disability and are strongly associated with negative health outcomes. Research suggests that 1 in 5 older adults with limitations in activities of daily living report needing more help than is received. The objective of this study is to address a gap in the literature by directly examining the relationship between adverse consequences (e.g. home-bound, bedridden) of unmet in-home mobility care needs and hospital stay for a national sample of community-dwelling older adults. Data was analyzed from round eight (2018) of the National Health and Aging Trends Study (NHATS), an epidemiologic panel study of nationally representative Medicare beneficiaries ages 65 and older living in the communities (n = 4,344). Community dwelling adults with one or more adverse consequence due to in-home mobility limitation had 1.931 times odds of hospital stay in the last 12 months, compared to the counterpart with no in-home mobility limitation (OR = 1.931, SE = 0.153, p < 0.05), after adjusting for the covariates. Community-dwelling older adults who have adverse consequence due to unmet in-home mobility care needs are more likely to be immobile and are more likely to have hospital stays. By addressing the needs of this population, the rate of hospitalization can be decreased resulting in fewer stressful events and better quality of life. Policies to improve long-term services and supports and reduce unmet need could benefit both older adults and those who care for them.

